# ﻿*Thailandorchestiarhizophila* sp. nov., a new genus and species of driftwood hopper (Crustacea, Amphipoda, Protorchestiidae) from Thailand

**DOI:** 10.3897/zookeys.1099.82949

**Published:** 2022-05-04

**Authors:** Koraon Wongkamhaeng, Pongrat Dumrongrojwattana, Ratchaneewarn Sumitrakij, Tosaphol Saetung Keetapithchayakul

**Affiliations:** 1 Department of Zoology, Faculty of Science, Kasetsart University, Bangkok, 10900, Thailand Kasetsart University Bangkok Thailand; 2 Department of Biology, Faculty of Science, Burapha University, Bangsaen, Chonburi, 20130, Thailand Burapha University Bangsaen Thailand; 3 National science museum, Khlong Ha, Khlong Luang District, Pathum Thani, 12120, Thailand National science museum Pathum Thani Thailand

**Keywords:** Description, Ko Kut District, marsh hopper, Talitroidea, *Thailandorchestia* gen. nov.

## Abstract

During a scientific survey, a new genus of driftwood hopper was found in mangrove roots in Ko Kut District, Trat Province, Thailand. We placed this new genus, *Thailandorchestia***gen. nov.**, within the family Protorchestiidae. The new genus can be distinguished from the remaining genera by uropod 1 outer ramus with robust setae, uropod 2 outer ramus without robust setae, and pereopod 7 basis without a posterodistal lobe. The type species of *Thailandorchestia***gen. nov.**, *Thailandorchestiarhizophila***sp. nov.**, is described herein, and an updated key to the genera of the family Protorchestiidae is provided.

## ﻿Introduction

The family Protorchestiidae is a mascupod family established by Myers and Lowry (2020) and contains 24 species belonging to six genera, namely *Cochinorchestia* Lowry & Peart, 2010, *Eorchestia* Bousfield, 1984, *Microrchestia* Bousfield, 1984, *Neorchestia* Friend, 1987, and *Protorchestia* Bousfield, 1982. All of them are classified as a post-Gondwanaland group (Myers and Lowry 2020). Each genus is distributed in different areas of the world, with *Cochinorchestia* located in southern India and Mozambique on the western coast of Africa (Lowry and Peart 2010; Lowry and Springthorpe 2015), *Eorchestia* in South Africa (Richardson 1993), and *Microchestia*, *Neochestia*, and *Protochestia* in Australia (Bousfield 1984; Friend 1987; Richardson 1996). All members of this group are marsh hoppers who occupy mangrove forests, except *Neorchestia*, which are forest hoppers. They all have some primitive characteristics, including: 1) maxilliped palp article 2 without a distomedial lobe; 2) article 4 small, distinct and gnathopod 2 subchelate; 3) pereopods 3–7 simplidactylate; and 4) pereopod 4 dactylus basidactylate.

Herein, we describe a 4-dentate noncuspidactylate palustral amphipod with basis of pereopod 7 without a posterodistal lobe as a new genus and species of the family Protorchestiidae. The new species was discovered in mangrove roots (*Rhizophora* sp.) and rotting logs in Ko Kut District, Trat Province, Thailand.

## ﻿Materials and methods

**Figure 1.**
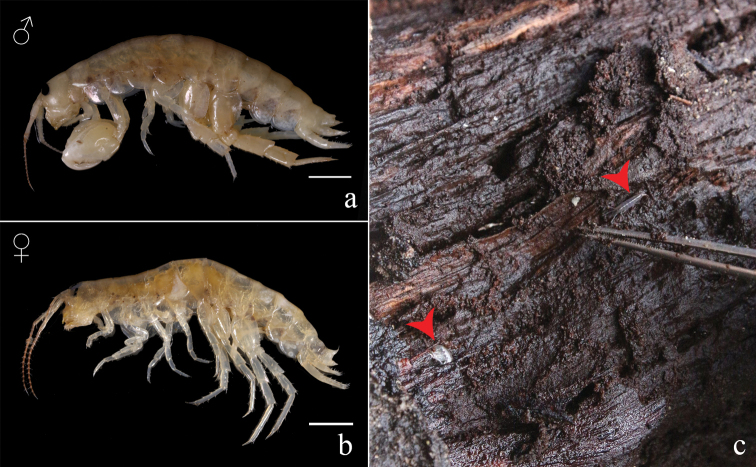
*Thailandorchestiarhizophila* sp. nov. **a** holotype, male, 8.04 mm, THNHM-Iv- 18760 **b** allotype, female, 7.80 mm, THNHM-IV- 18961 **c** rotting mangrove log, habitat of *Thailandorchestiarhizophila* sp. nov.

Amphipods were collected from driftwood, rotting logs and mangrove roots (*Rhizophora* sp.) in a mangrove forest near Ao Phrao, Ko Kut District, Trat Province, Thailand (11°35'40.2"N, 102°33'52.6"E) (Fig. 1). The mangrove forest is located near a small creek 50 meters from the beach. Twelve rotting logs were broken apart and 15–30 amphipod individuals were found inside each log (see Suppl. material 1). The amphipod specimens were sorted and fixed in 70% ethanol. The specimens were transferred from ethanol onto a glycerol slide for morphological study in the laboratory. Drawings were made using a drawing tube attached to an Olympus CH30 light microscope. The pencil drawings were scanned and digitally inked using a WACOM bamboo CTH-970 graphics board in Adobe Illustrator CC 2017, following the method described in Coleman (2003). Setae and mouthparts were following Zimmer et al. (2009). Abbreviations used in the text are as follows: A, antenna; G, gnathopod; UL, labrum; LL, labium; MD, mandible; MX, maxilla; MP, maxilliped; P, pereopod; p, palp; pl, pleopod; T, telson; U, uropod; L, left; R, right. Institutional abbreviations: THNHM, Thailand Natural History Museum, Bangkok, Thailand.

## ﻿Results

### ﻿Systematics

**Order Amphipoda Latreille, 1816**

**Suborder Senticaudata Lowry & Myers, 2013**

#### Family Protorchestiidae Myers & Lowry, 2020

##### ﻿Genus Thailandorchestia gen. nov.

Taxon classificationAnimaliaAmphipodaProtorchestiidae

FAEC6373-5F05-5457-B399-40AAEDB21B44

http://zoobank.org/9DDD49ED-997C-430E-B06A-2586B8DB34EE

###### Type species.

*Thailandorchestiarhizophila* sp. nov., here designated.

###### Diagnosis.

Protorchestiidae with ***maxilliped*** palp article 2 distomedial lobe absent. ***Mandible*** left lacinia mobilis 4-dentate. ***Gnathopod 2*** coxal gill simple. ***Pereopod 4*** carpus significantly shorter than carpus of pereopod 3. ***Pereopods 6–7*** sexually dimorphic (male merus and carpus incrassate). ***Pereopod 7*** posterodistal lobe absent. ***Uropod 1*** peduncle distolateral robust setae present, very large (1/3–1/2 length of outer ramus); inner ramus linear, not modified; outer ramus with marginal robust setae. ***Uropod 2*** outer ramus without marginal robust setae. ***Uropod 3*** peduncle with 2 robust setae; ramus shorter than peduncle, linear (narrowing). ***Telson*** apically incised, with 2 robust setae per lobe.

###### Etymology.

The generic name, *Thailandorchestia* gen. nov., is derived from “Thailand” in combination with the *Orchestia* stem.

###### Type locality.

Mangrove forest near Ban Ao Prao Beach (11°35'40.2"N, 102°33'52.6"E), Trat Province, Thailand.

###### Ecological type.

Driftwood hoppers (virtually confined to rotting driftwood where they live in galleries, consuming rotting driftwood and reproducing with relatively small broods).

###### Remarks.

The new genus clearly belongs to Protorchestiidae due to the presence of: 1) maxilliped palp article 2 without distomedial lobe; 2) article 4 small, distinct; 3) gnathopod 2 subchelate; 4) pereopods 3–7 simplidactylate; 5) pereopod 4 dactylus basidactylate; and 6) telson with apical robust setae only or with apical and marginal robust setae, with 1–6 robust setae per lobe.

The new genus is closely related to *Microrchestia* in having: 1) left mandible larcinia mobilis 4-dentate; 2) carpus of pereopod 3 subequal to those of pereopod 4; and 3) pereopods 6 and 7 sexually dimorphic. However, the current genus differs from *Microrchestia* from Australia by having: 1) maxilliped palp article 2 distomedial lobe absent (vs. well developed); 2) pereopod 7 posterodistal lobe absent (vs. present), and 3) U1 outer ramus with marginal robust setae (vs. without marginal robust setae) (Table 1).

**Table 1.** Comparison of diagnostic characteristics in different protorchestiid genera.

Genus	MP palp article 2 distomedial lobe	LMD lacinia mobilis	G1 sexual dimorphism	Carpi of P4:P3	P6–7 sexual dimorphism	P7 postero- distal lobe	U1 outer ramus	U2 outer ramus marginal setae	U3 robust setae on peduncle	U3 ramus	Number of setae per telsonic lobe
*Carpentaria*	well developed	4-dentate	absent, palm obtuse	subequal	absent	present	linear without marginal setae	present	1–4	bud-like	3–6
*Cochinorchestia*	present	4-dentate	absent, palm transverse	longer	unknown	present	spoon-shape with marginal setae	absent	1	linear	2
*Eorchestia*	absent	4-dentate	absent, palm transverse	longer	absent	present	linear without marginal setae	absent	3	linear	1–2
*Microrchestia*	well developed	4-dentate	present, palm transverse	longer	present	present	linear without marginal setae	absent	2	linear	2
*Neorchestia*	absent	5-dentate	absent, palm transverse	longer	unknown	present	linear without marginal setae	absent	2	linear	1
*Protorchestia*	absent	5-dentate	absent, palm transverse	subequal	absent	present	linear without marginal setae	absent	3	linear	2
*Thailandorchestia* gen. nov.	absent	4-dentate	present, palm transverse	longer	present	absent	linear with marginal setae	absent	2	linear	2

Only one protochestiid amphipod had been previously reported from Thailand. Bussarawich (1985) studied the diversity of amphipods in the mangrove forest and reported *Microchestia* sp., a member of the family Protorchestiidae. Later, Lowry and Springthorpe (2015) revised the genus *Cochinorchestia* Lowry & Peart, 2010. Although the *Microrchestia* sp. from Thailand was also mentioned as a *Cochinorchestia* sp. based on the illustration of the previous publication, some details such as the maxilliped and gnathopods 1 and 2 remain unclear. The specimens from the report of Bussarawich (1985) presumed lost, which makes the *Cochinorchestia* sp. in this report still tentative.

The new genus is similar to *Cochinorchestia* from China in having: 1) left mandible larcinia mobilis 4-dentate; 2) carpus of pereopod 3 longer than that of pereopod 4; and 3) uropod 1 outer ramus with marginal setae. However, the current genus differs from *Cochinorchestia* in having: 1) pereopod 7 without a posterodistal lobe (vs. pereopod 7 with a posterodistal lobe); 2) uropod 1 outer ramus linear (vs. spoon-shaped) and uropod 3 peduncle with 2 robust setae (vs. with 3 robust setae); and 3) uropod 2 outer ramus without robust setae (vs. with marginal robust setae in 1 row).

The new genus is identifiable using the following key to genera of Protorchestiidae.

### ﻿Key to genera of Protorchestiidae

**Table:** 

1	Uropod 3 peduncle with 4 robust setae	***Carpentaria***
–	Uropod 3 peduncle with less than 4 robust setae	**2**
2	Uropod 3 peduncle with 3 robust setae	**3**
–	Uropod 3 peduncle with less than 3 robust setae	**4**
3	Maxilliped palp article 2 distomedial lobe absent; mandible left lacinia mobilis 4-dentate; pereopod 4 carpus shorter than carpus of pereopod 3	***Eorchestia***
–	Maxilliped palp article 2 distomedial lobe present; mandible left lacinia mobilis 5-dentate; pereopod 4 carpus subequal to carpus of pereopod 3	***Protorchestia***
4	Uropod 3 peduncle with 1 robust seta	***Cochinorchestia***
–	Uropod 3 peduncle with 2 robust setae	**5**
5	Mandible left lacinia mobilis 5-dentate	***Neorchestia***
–	Mandible left lacinia mobilis 4-dentate	**6**
6	Uropod 1 outer ramus without marginal robust setae; basis of pereopod 7 with a posterodistal lobe	***Microrchestia***
–	Uropod 1 outer ramus with marginal robust setae; basis of pereopod 7 without a posterodistal lobe	***Thailandorchestia* gen. nov.**

#### ﻿ Thailandorchestia rhizophila sp. nov.

Taxon classificationAnimaliaAmphipodaProtorchestiidae

60E73045-2940-5CB0-A546-C5FCDCB224E0

http://zoobank.org/BDA296BD-ED94-4AEA-AF3E-B6894581D459

##### Diagnosis.

As for the genus unless otherwise stated. ***Antenna 1*** long, reaching from midpoint to end of article 5 of antenna 2 peduncle. ***Eye*** medium (1/5–1/3 of head length). ***Gnathopod 1*** not sexually dimorphic, palm transverse, dactylus shorter than palm. ***Gnathopod 2*** sexually dimorphic (male subchelate, female mitten-shaped). ***Pleopod 1*** outer ramus subequal in length to peduncle. ***Pleopod 3*** outer ramus longer than peduncle.

##### Material examined.

***Holotype***, male, 8.04 mm, THNHM-Iv- 18760; allotype, female, 7.80 mm, THNHM-IV- 18961; ***Paratypes***, 2 males, 1 non-gravid female, and 2 gravid females, THNHM- Iv 18761. All collected from the type locality on 4 May 2019, KW and PD leg.

##### Ecology.

Driftwood hoppers, living inside rotten logs and mangrove roots in the softest part under the bark. The mangrove forest is located near a small creek 50 meters from the beach. The sediment in the forest is muddy sand mixed with leaf litter.

##### Type locality.

Mangrove forest near Ban Ao Prao Beach (11°35'40.2"N, 102°33'52.6"E), Ko Kut District, Trat Province, Thailand.

##### Etymology.

The specific epithet refers to the habitat of this amphipod, which is also found inside mangrove roots.

##### Description of male holotype.

**Figure 2.**
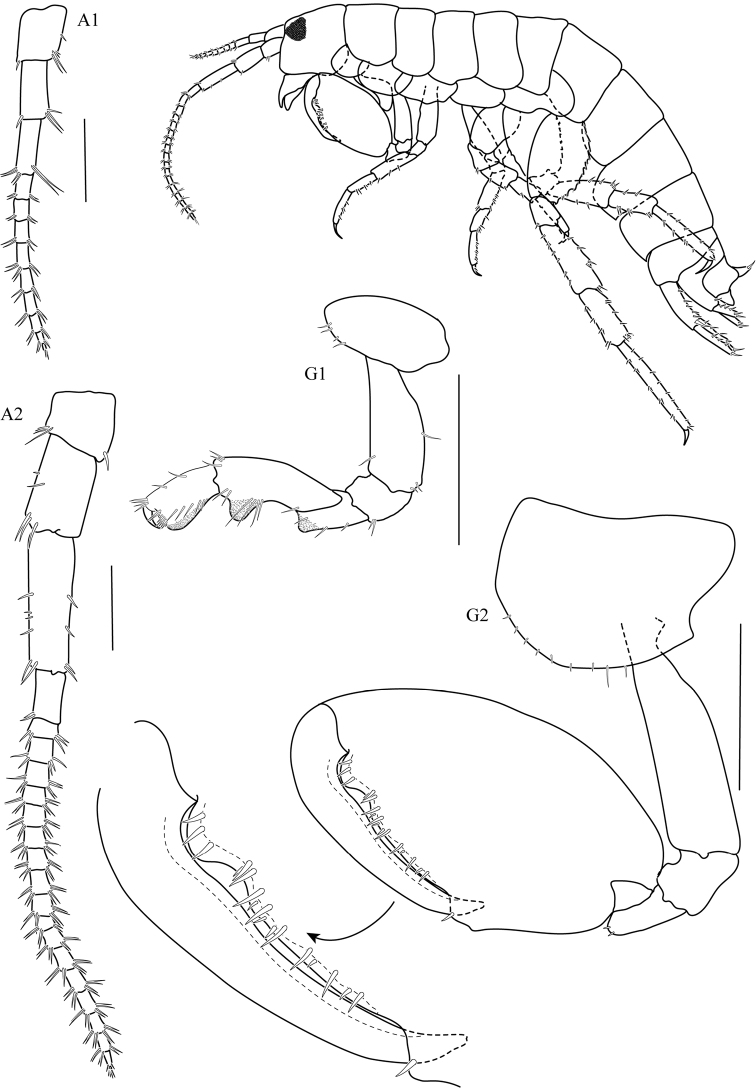
*Thailandorchestiarhizophila* sp. nov. holotype, male, 8.04 mm, THNHM-Iv- 18760. Scale bars: 1 mm.

**Figure 3.**
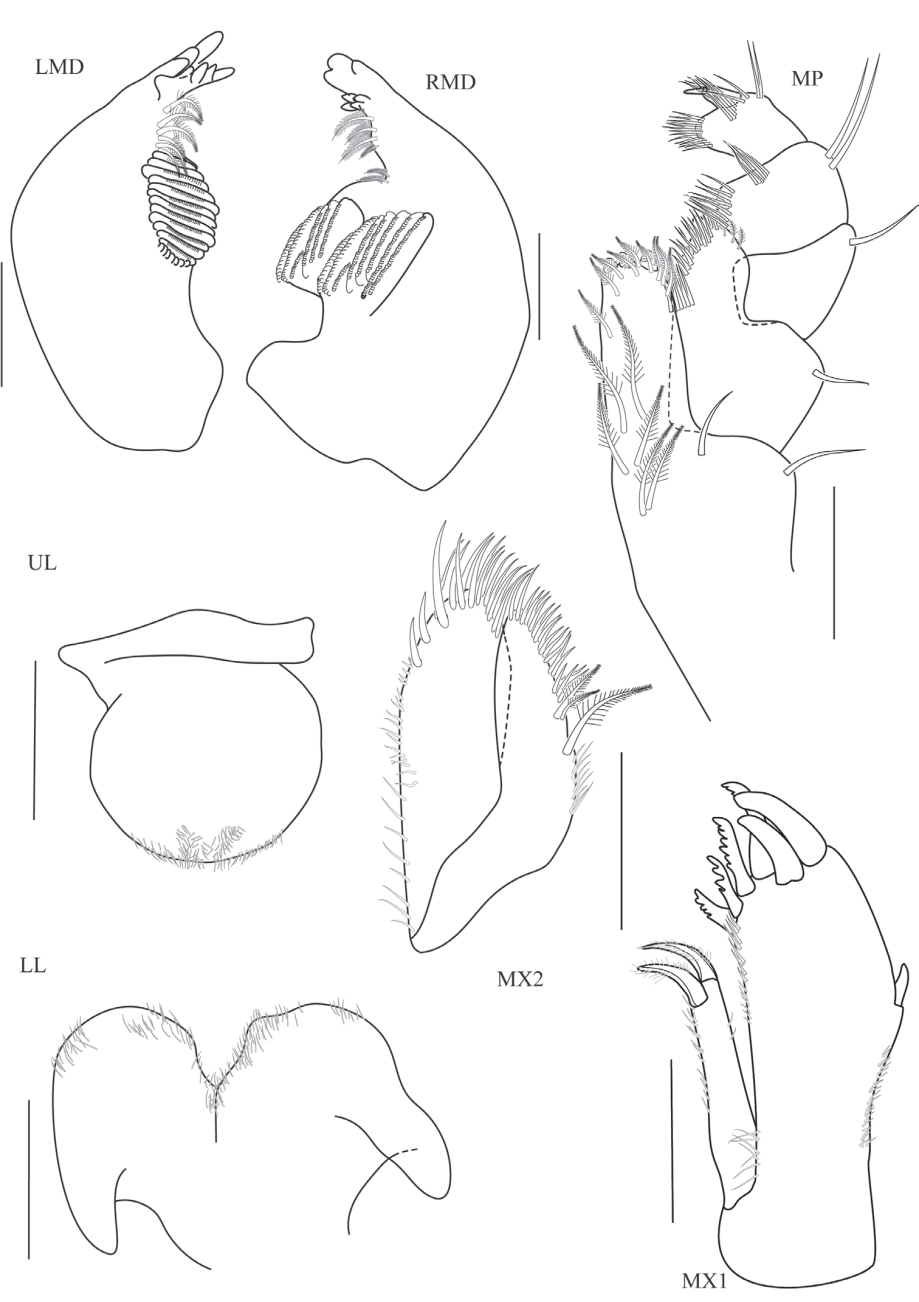
*Thailandorchestiarhizophila* sp. nov. holotype, male, 8.04 mm, THNHM-Iv- 18760. Scale bars: 0.2 mm.

**Figure 4.**
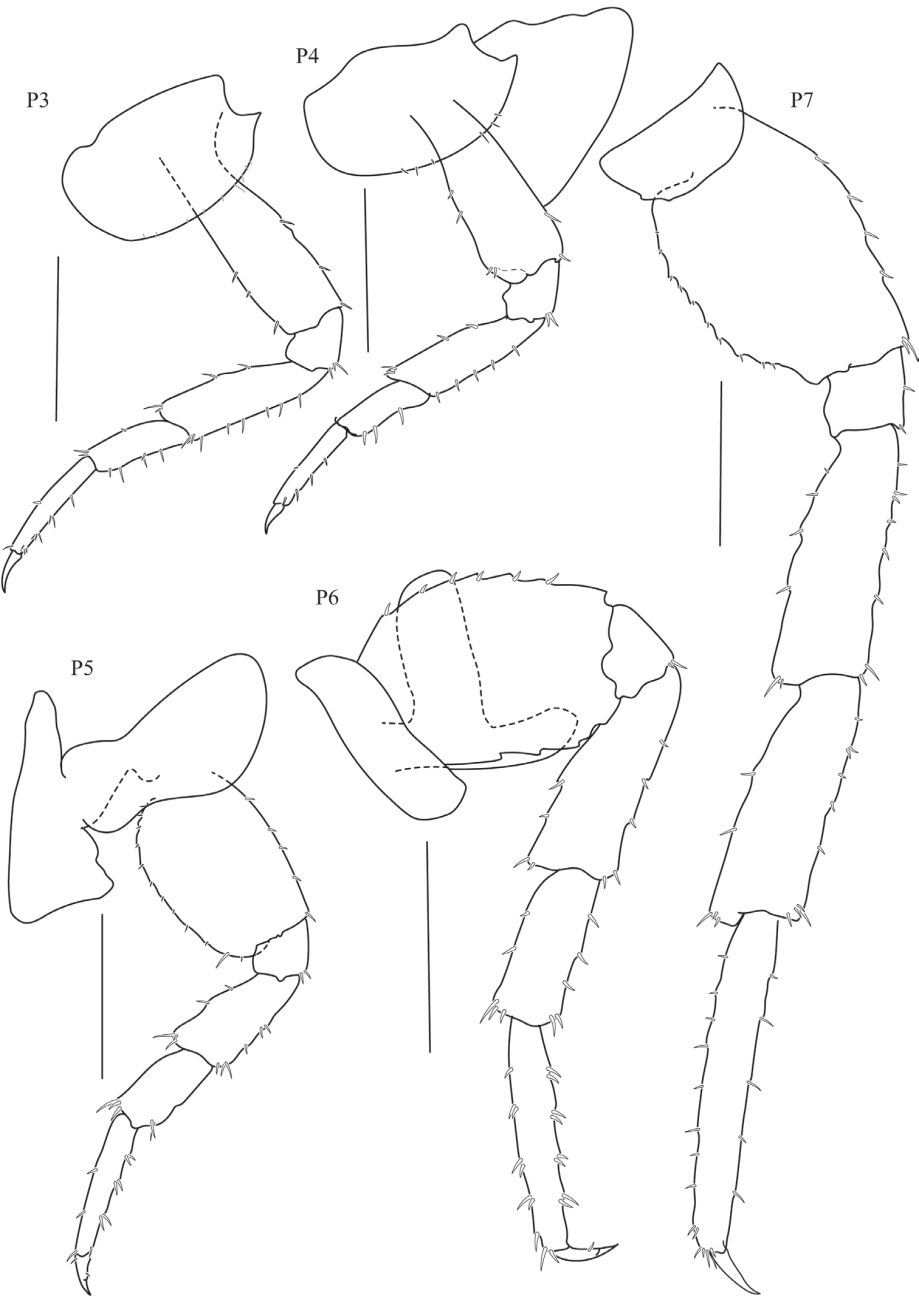
*Thailandorchestiarhizophila* sp. nov. holotype, male, 8.04 mm, THNHM-Iv- 18760. Scale bars: 1 mm.

**Figure 5.**
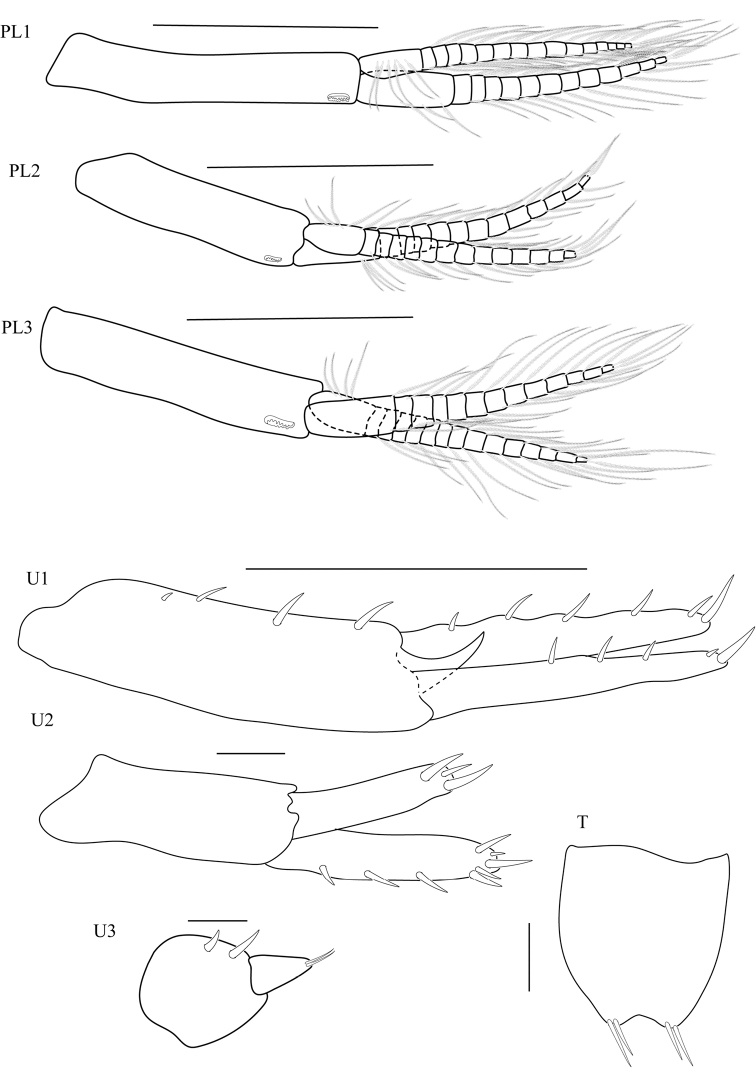
*Thailandorchestiarhizophila* sp. nov. holotype, male, 8.04 mm, THNHM-Iv- 18760. Scale bars (U1–U3, T): 0.1 mm; (PL1–PL3): 0.5 mm.

(THNHM-Iv- 18760, Figs 2–5).

**Head. *Eye*** medium (1/5–1/3 head length). ***Antenna 1*** (Fig. 2A1) long, reaching from midpoint to end of article 5 of antenna 2 peduncle. ***Antenna 2*** (Fig. 2A2) peduncular articles slender, article 5 longer than article 4. ***Upper lip*** (Fig. 3UL) without robust setae. ***Mandible*** (Fig. 3LMD) left lacinia mobilis 4-dentate. ***Maxilla 1*** (Fig. 3MX1) with small palp, 1-articulate. ***Maxilliped*** (Fig. 3MP) palp article 2 distomedial lobe absent; article 4 small, well defined.

**Pereon. *Gnathopod 1*** (Fig. 2G1) sexually dimorphic; subchelate; coxa 1 smaller than coxa 2; posterior margins of merus, carpus, and propodus each with lobe covered in palmate setae, palmate lobes present; propodus shorter than carpus, subrectangular; palm transverse. ***Gnathopod 2*** (Fig. 2G2) sexually dimorphic; subchelate; coxal gill simple (or slightly lobate); basis slender; carpus triangular, reduced (enclosed by the merus and propodus), posterior lobe absent, not projecting between merus and propodus; 1.8× as long as wide; palm acute, weakly toothed, with a subquadrate protuberance near dactylar ringe, lined with robust setae, posterodistal corner with socket; dactylus subequal in length to palm. ***Pereopod 3–4*** (Fig. 4P3–P4) coxae wider than deep. ***Pereopods 3–7*** (Fig. 4P3–P7) simplidactylate. ***Pereopod 4*** (Fig. 4P4) subequal or slightly shorter than pereopod 3; carpus similar in length to pereopod 3 carpus; dactylus similar to pereopod 3 dactylus. ***Pereopod 5*** propodus distinctly longer than carpus. ***Pereopod 6*** (Fig. 4P6) slightly sexually dimorphic; shorter than pereopod 7; coxa posterior lobe inner view posteroventral corner rounded, posterior margin oblique with respect to ventral margin, posterior lobe without a ridge, posterior lobe without marginal setae; coxal gill lobate. ***Pereopod 7*** (Fig. 4P7) sexually dimorphic (merus and carpus broadly incrassate); basis lateral sulcus absent, posterodistal lobe absent; distal articles (merus and carpus) expanded; merus posterior margin expanded distally, subtriangular.

**Pleon. *Pleopods*** all well developed. ***Pleopod 1*** (Fig. 5PL1) peduncle without marginal setae; biramous, outer ramus subequal in length to peduncle; inner ramus with 17 articles, outer ramus with 13 articles. ***Pleopod 2*** (Fig. 5PL2) peduncle without marginal setae; biramous, outer ramus subequal in length to peduncle; inner ramus with 15 articles, outer ramus with 14 articles. ***Pleopod 3*** (Fig. 5PL3) peduncle without marginal setae; biramous, outer ramus subequal in length to peduncle; inner ramus with 15 articles, outer ramus with 13 articles. ***Uropod 1*** (Fig. 5U1) peduncle with 4 robust setae, distolateral robust seta present, large (1/4 length of outer ramus), with simple tip; inner ramus subequal in length to outer ramus, inner ramus with marginal robust setae; outer ramus with 3 marginal robust setae. ***Uropod 2*** (Fig. 5U2) inner ramus subequal in length to outer ramus, with marginal robust setae, with 3 lateral robust setae; outer ramus without marginal robust setae. ***Uropod 3*** (Fig. 5U2) peduncle with 2 robust setae; ramus shorter than peduncle, ramus triangular, with 2 apical setae. ***Telson*** (Fig. 5T) longer than broad, apically incised, dorsal midline vestigial or absent, with apical robust setae only and 2 robust setae per lobe.

##### Description of female allotype.

**Figure 6.**
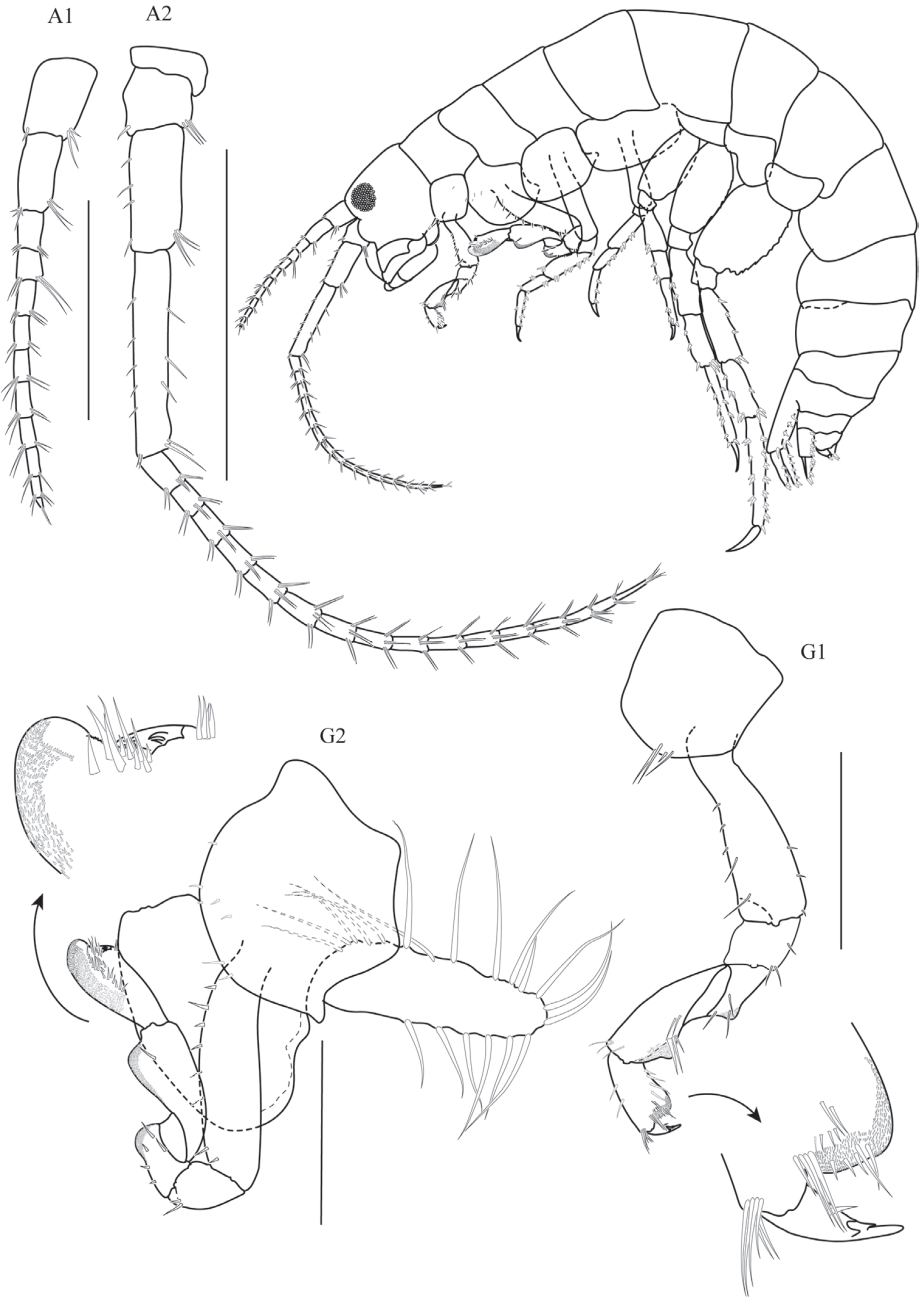
*Thailandorchestiarhizophila* sp. nov. allotype, female, 7.80 mm, THNHM-Iv- 18761. Scale bars: 1 mm.

**Figure 7.**
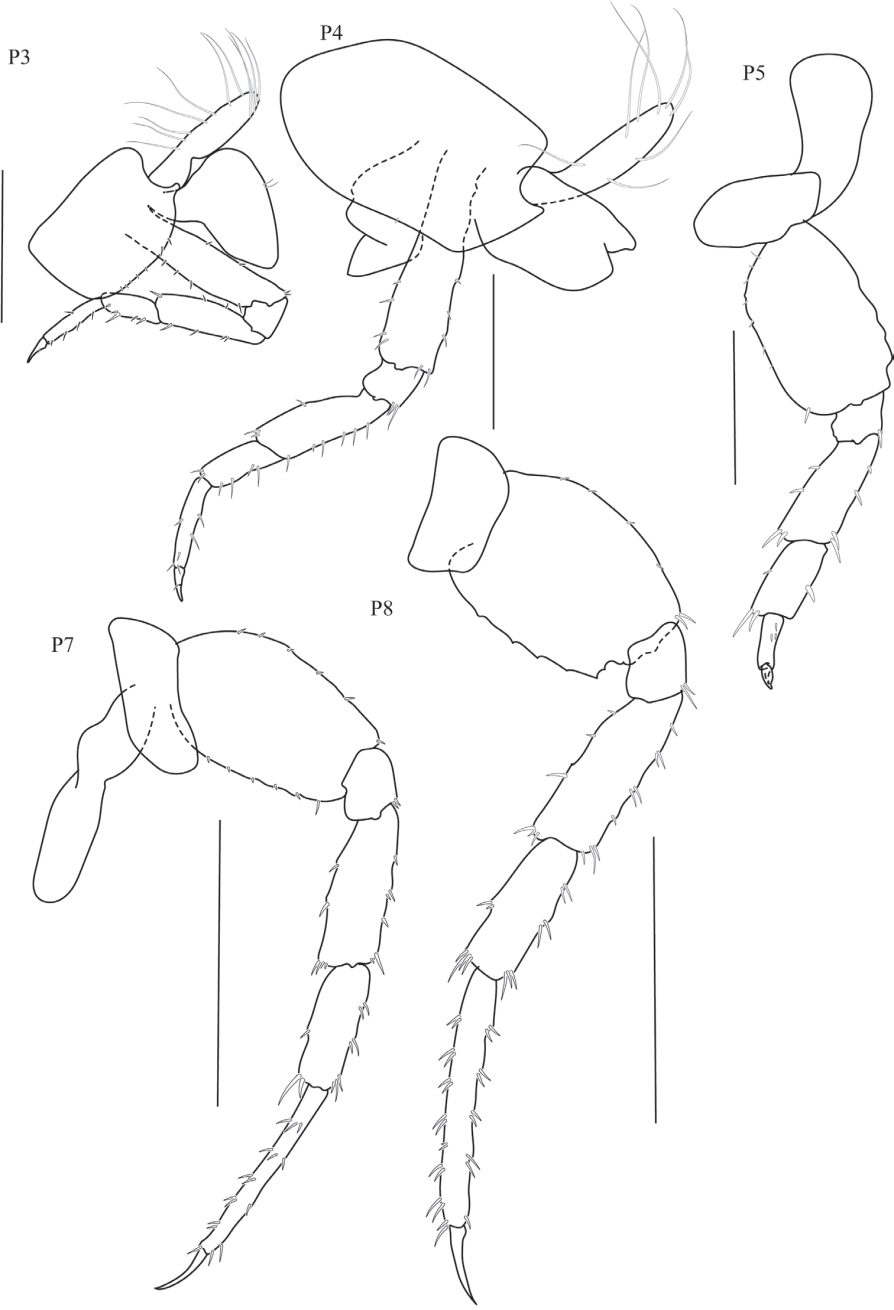
*Thailandorchestiarhizophila* sp. nov. allotype, female, 7.80 mm, THNHM-Iv- 18761. Scale bars: 1 mm.

(THNHM-Iv- 18761, Figs 6–7)

**Pereon**. ***Gnathopod 1*** (Fig. 6G1) propodus narrower than that of male; dactylus subequal to palm. ***Gnathopod 2*** (Fig. 6G2) mitten-shaped; basis slightly expanded; posterior margins of merus, carpus, and propodus each with lobe covered in palmate setae; carpus well developed (not enclosed by merus and propodus), posterior lobe present, projecting between merus and propodus; propodus length twice as long as wide; palm obtuse, smooth, without a protuberance or shelf near dactylar hinge, posterodistal corner naked; dactylus shorter than palm; gill lobate. ***Pereopod 5*** (Fig. 7P5) propodus shorter than carpus. Distal articles (merus and carpus) slender. ***Preopods 6–7*** (Fig. 7P6–7) sexually dimorphic (merus and carpus not broadly incrassate). ***Oostegites*** long (length greater than 2× width), longer than wide, weakly setose, setae with simple, smooth tips.

**Figure 8.**
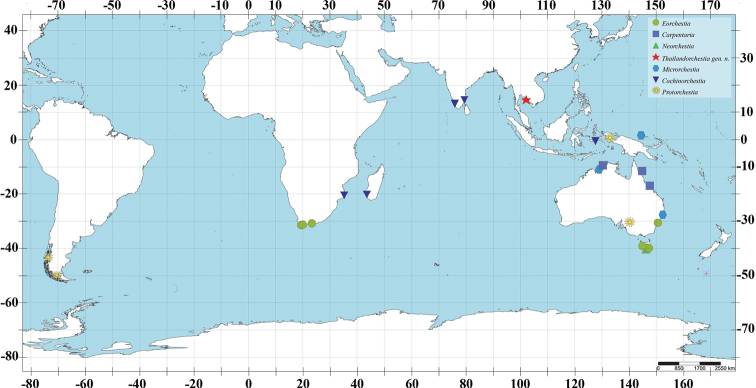
Map showing distribution of genera in the family Protorchestiidae.

##### Habitat.

Mangrove wood, inside roots and rotting logs.

##### Distribution.

Thailand, Ko Kut District, Inner Gulf of Thailand.

## ﻿Discussion

Most species of Protorchestiidae are known to be semiaquatic marsh hoppers that inhabit salt marshes and mangrove swamps (Myers and Lowry 2020), except for *Neorchestia*, which has adapted to life on land (Friend 1987). Protochestiid amphipods were previously reported to live in hard substrates (rock) and soft substrates (sand, mangrove debris, and wet forest soil) (Myers and Lowry 2020). Surprisingly, *Thailandorchestia* gen. nov. specimens live in galleries inside the mangrove roots, where gravid females are also found, implying that these amphipods reproduce inside the roots. According to this ecology, these amphipods should be classified as driftwood hoppers. This is the second genus reported as a driftwood hopper; a previous driftwood hopper report is of the genus *Macarorchestia* in the northeast Atlantic and Mediterranean coastal regions (Wildish 2014). Based on these observations, the adaptations observed in *Thailandorchestiarhizophila* sp. nov. are akin to those in *Macarorchestia* in having: 1) reduced pleopod and oostegites; 2) fewer ova per brood (5–6 individuals); 3) small eyes; and 4) lack of dorsal pigment (Wildish 2017). Another behavior found in the present study was negative phototaxis, whereby *T.rhizophila* sp. nov. specimens escaped deeper inside the wood upon its splitting.

According to the recent checklist of the amphipods of Southeast Asia (Azman et al. 2022), a total of 25 species of Talitroidea amphipods have been reported, with four species (16%) occurring in Thailand. From that, *Thailandorchestiarhizophila* sp. nov. is the only one species has been reported from mangrove forest while consider the area of mangrove forest in Thailand covers 2,300 square kilometres (Pumijumnong 2014). Further intensive study of mangrove amphipods, especially in the marsh hopper group, is required.

## Supplementary Material

XML Treatment for Thailandorchestia

XML Treatment for Thailandorchestia rhizophila

## Supplementary materials

Supplementary material 1 Video of living *Thailandorchestiarhizophila* Data type Mp4 file. Explanation note Video of living *Thailandorchestiarhizophila* inside the rotting branch and their food item. https://binary.pensoft.net/file/680166This dataset is made available under the Open Database License (http://opendatacommons.org/licenses/odbl/1.0/). The Open Database License (ODbL) is a license agreement intended to allow users to freely share, modify, and use this Dataset while maintaining this same freedom for others, provided that the original source and author(s) are credited.Koraon Wongkamhaeng, Pongrat Dumrongrojwattana, Ratchaneewarn Sumitrakij, Tosaphol Saetung Keetapithchayakul

## References

[B1] Azman AR Sivajothy K Shafie BB Ja’afar N Wongkamhaeng K Bussarawit S Alip AE Lee YL Metillo EB Won MEQ (2022) The amphipod (Crustacea: Peracarida) of the Southeast Asia and the neighbouring waters: an updated checklist with new records of endemic species. Research Bulletin - Phuket Marine Biological Center 79(1): 42–84.

[B2] Bousfield EL (1982) The amphipod superfamily Talitroidea in the northeastern Pacific region. Family Talitridae. Systematics and distributional ecology. National. Museum of Natural Science, Publications in Biological Oceanography, 11, [i–vii] 73 pp.

[B3] Bousfield EL (1984) Recent advances in the Systematics and Biogeography of Landhoppers (Amphipoda: Talitridae) of the Indo-Pacific Region. Bishop Museum Special Publications 72: 169–205.

[B4] Bussarawich S (1985) Amphipod Mangroves Thailand. In: NRCT (Ed.) Fifth Seminar on Mangrove Ecosystems, Phuket, Thailand, 17 pp.

[B5] Coleman C (2003) “Digital inking”: How to make perfect line drawings on computers. Organisms, Diversity & Evolution 3(4): 1–14. 10.1078/1439-6092-00081

[B6] Friend JA (1987) Terrestrial Amphipods (Amphipoda: Talitridae) of Tasmania: Systematics and Zoogeography. Records of the Australian Museum 7: 1–85. 10.3853/j.0812-7387.7.1987.97

[B7] Latreille PA (1816) Nouveau Dictionnaire d’histoire naturelle, appliquée aux arts, à l’Agriculture, à l’Economic rurale et domestique, à la Médecine, etc. Par une Société de Naturalistes et d’Agriculteurs. Nouvelle Édition. Paris. 1: 467–469.

[B8] Lowry JK Peart R (2010) The genus *Microrchestia* (Amphipoda: Talitridae) in eastern Australia. Zootaxa 2349(1): 23–38. 10.11646/zootaxa.2349.1.2

[B9] Lowry JK Springthorpe RT (2015) Coastal Talitridae (Amphipoda: Talitroidea) from north-western Australia to Darwin with a revision of the genus *Cochinorchestia* Lowry & Peart, 2010. Zootaxa 3985(2): 151–202. 10.11646/zootaxa.3985.2.1 26250029

[B10] Myers AA Lowry JK (2020) A phylogeny and classification of the Talitroidea (Amphipoda, Senticaudata) based on interpretation of morphological synapomorphies and homoplasies. Zootaxa 4778(2): 281–310. 10.11646/zootaxa.4778.2.3 33055821

[B11] Pumijumnong N (2014) Mangrove Forests in Thailand. In: Faridah-Hanum I Latiff A Hakeem K Ozturk M (Eds) Mangrove Ecosystems of Asia.Springer, New York, NY, New York, 60–79. 10.1007/978-1-4614-8582-7_4

[B12] Richardson AMM (1993) Tasmanian intertidal Talitridae (Crustacea: Amphipoda). Palustral talitrids: two new species of *Eorchestia* Bousfield, 1984. Journal of Natural History 27(2): 267–284. 10.1080/00222939300770131

[B13] Richardson AMM (1996) *Protorchestialakei*, new species (Amphipoda: Talitridae), from Maatsuyker Island, Tasmania, with a key to the genus and notes on the diversity of Tasmanian Talitridae. Journal of Crustacean Biology 16(3): 574–583. 10.2307/1548749

[B14] Wildish DJ (2014) New genus and two new species of driftwood hoppers (Crustacea, Amphipoda, Talitridae) from northeast Atlantic and Mediterranean coastal regions. Zoosystematics and Evolution 90(2): 133–146. 10.3897/zse.90.8410

[B15] Wildish DJ (2017) Evolutionary ecology of driftwood talitrids: A review. Zoosystematics and Evolution 93(2): 353–361. 10.3897/zse.93.12582

[B16] Zimmer A Araujo PB Bond-Buckup G (2009) Diversity and arrangement of the cuticular structures of *Hyalella* (Crustacea: Amphipoda: Dogielinotidae) and their use in taxonomy. Zoologia 26(1): 127–142. 10.1590/S1984-46702009000100019

